# Concatenation and Species Tree Methods Exhibit Statistically Indistinguishable Accuracy under a Range of Simulated Conditions

**DOI:** 10.1371/currents.tol.34260cc27551a527b124ec5f6334b6be

**Published:** 2015-03-09

**Authors:** João Tonini, Andrew Moore, David Stern, Maryia Shcheglovitova, Guillermo Ortí

**Affiliations:** Department of Biological Sciences, The George Washington Univerisity, Washington, District of Columbia, USA; Department of Biological Sciences, The George Washington University, Washington, District of Columbia, USA; Computational Biology Institute, Department of Biological Sciences, The George Washington University, Washington, District of Columbia, USA; Department of Geography & Environmental Systems, University of Maryland Baltimore County, Baltimore, MD, USA; Department of Biological Sciences, The George Washington Univerisity, Washington, District of Columbia, USA

## Abstract

Phylogeneticists have long understood that several biological processes can cause a gene tree to disagree with its species tree. In recent years, molecular phylogeneticists have increasingly foregone traditional supermatrix approaches in favor of species tree methods that account for one such source of error, incomplete lineage sorting (ILS). While gene tree-species tree discordance no doubt poses a significant challenge to phylogenetic inference with molecular data, researchers have only recently begun to systematically evaluate the relative accuracy of traditional and ILS-sensitive methods. Here, we report on simulations demonstrating that concatenation can perform as well or better than methods that attempt to account for sources of error introduced by ILS. Based on these and similar results from other researchers, we argue that concatenation remains a useful component of the phylogeneticist’s toolbox and highlight that phylogeneticists should continue to make explicit comparisons of results produced by contemporaneous and classical methods.

## Introduction

For decades, phylogeneticists have recognized the potential for discordance between a gene tree and its species tree 1, ^,^ 2. Maddison’s seminal insights on the topic 3 illustrate how discordance may arise from any of the following sources: gene duplication and extinction (“hidden paralogy”), lateral gene transfer, and incomplete lineage sorting (ILS). Given the computational and theoretical difficulties of adequately modeling gene descent, phylogeneticists have traditionally not attempted to reconstruct species phylogenies with models that explicitly account for sources of gene trees-species tree discord, and have instead opted to increase the total number of loci included in their concatenated data matrices under the expectation that, on average, the signal of multiple genes will recover the true species tree.

In recent years, however, molecular systematists have begun eschewing traditional supermatrix methods in favor of explicit modeling of the processes that may generate discordance. Although issues related to gene duplication and lateral gene transfer have received some attention 4, ^,^ 5, most efforts have concentrated on accounting for the effects of ILS by modeling the multispecies coalescent 6. The barrage of new methods now available to account for error due to ILS (Table 1) and the general transition away from supermatrix methods seem to follow the predicted emergence of a new paradigm in molecular systematics 7.

**Table 1. Species tree methods accounting for incomplete lineage sorting in molecular systematics:** Note that this list is not exhaustive.

Method	Details
**BATWING** 8	Uses Metropolis–Hastings algorithms to estimate population histories and mutation rates. The ancestral DNA data are inferred at each node of the genealogical tree. Different types of sequence data can be analyzed. The program estimates demographic parameters, but do not allow for the effects of either recombination or selection.
*BEAST 6	Explicitly models the multispecies coalescent, coestimating multiple gene trees within a shared species tree and effective population sizes of extant and extinct populations in a Bayesian Markov chain Monte Carlo framework.
**BEST** 9, ^,^ 10, ^,^ 11	Estimates species tree topology, divergence times, and population sizes from gene trees under a multispecies coalescent model in a Bayesian framework; unlike *BEAST, BEST estimates each gene tree individually, then uses importance sampling to infer the species tree.
**BUCKy** 12	Uses non-parametric clustering of genes to estimate concordance factors based on proportions of genes supporting a clade; these factors provide the basis for inferring species-trees. Implemented in a Bayesian phylogenetic framework, and thus integrates over gene tree uncertainty.
**GLASS** 13	An estimator of pairwise divergence times under the multispecies coalescent. Applies pairwise estimates by single-linkage clustering to generate species trees with branch lengths, using multiple unlinked loci and allow the use of several alleles from each population.
**iGLASS** 14	Improves GLASS estimation of divergence times by deriving the expected waiting time until the first interspecific coalescence occurs among independent loci for a pair of taxa.
**MCMCcoal** 15	A Bayes method for simultaneous estimation of the species divergence times and current and ancestral population sizes. Uses multiple loci, the topology of the species tree is assumed known, and a MCMC algorithm integrates over uncertain gene trees and branch lengths (or coalescence times) at each locus as well as species divergence times. It can handle any species tree and allows different numbers of sequences at different loci.
**MDC** 16	Parsimony-based method that heuristically searches for the species tree that minimizes the number of deep coalescences implied by the gene trees, and thus does not model the coalescent process.
**MP-EST** 17	Uses pseudo-likelihood function of the species tree to obtain maximum pseudo-likelihood estimates of species trees, with branch lengths in coalescent units. It assumes no gene flow or horizontal gene transfer, but method is robust to a small amount of the former.
**NJst** 18	The distance between two species is defined as the average number of internodes between two species across gene trees, then the species tree is estimated by the neighbor joining tree built from the distance matrix.
**SNAPP** 19	Uses a polynomial-time algorithm that computes the likelihood of a species tree directly from the sequences under a finite-sites model of mutation effectively integrating over all possible gene trees. The method applies to unlinked biallelic markers and it is implemented in a Markov chain Monte Carlo sampler for inferring species trees, divergence dates, and population sizes.
**STAR** 20	Uses summary statistics of coalescence times by ordering the expected ranks of the coalescences among sequences, which is consistent with the ancestral order of populations in the species tree. It is resistant to variable substitution rates along the branches in gene trees.
**STEAC** 20	Uses summary statistics of average coalescence times to estimate the species tree. The gene trees are generated from multilocus sequences using a consistent method for gene tree estimation (e.g. maximum likelihood) without the molecular clock assumption.
**STEM** 21	Analytically derives the maximum likelihood species tree for a set of gene trees with branch-length information, explicitly modeling discord as a function of the coalescent process.
**SVDquartets** 22	Infers relationships among quartets of taxa under the coalescent model using techniques from algebraic statistics; it accounts for mutational and coalescent variance. Uncertainty in the estimated relationships is quantified using the nonparametric bootstrap.
**STELLS** 23	Uses coalescent-based maximum likelihood approach for inferring the species tree from a set of gene tree topologies (no branch lengths), which are used to estimated branch lengths in coalescent units.; the algorithm assumes free recombination between genes. It assumes the gene tree topologies are correctly inferred, therefore does not model uncertainty of gene tree estimation from sequences.

Inadequate model specification is known to introduce systematic bias into phylogenetic analyses, and thus the drive to account for potential error introduced by ILS has merit on theoretical grounds 16, ^,^ 24, ^,^ 25. However, the conditions under which traditional supermatrix approaches to molecular systematics necessarily result in less accurate topologies than methods accounting for population genetic processes remain unresolved, and recent empirical studies have reported high congruence between supermatrix and species tree methods 26, ^,^ 27, ^,^ 28.

Moreover, while theoretical studies have demonstrated the existence of species trees for which discordant gene trees are more likely than genealogies that agree with the species tree (the “anomalous gene trees” [AGTs] of Degnan and Rosenberg 24), such species histories are apparently unlikely to mislead most phylogenetic analyses. Huang and Knowles 29 have shown that, for species trees containing anomaly zones (i.e. short internal branches that result in AGTs), the probability of observing a polytomous gene tree is greater than the probability of recovering an anomalous gene tree. Thus, for species trees with short, deep branches, gene trees are more likely to be uninformative than to be outright misleading.

Given such empirical and theoretical findings, traditional supermatrix approaches may still be adequate for densely sampled data matrices and for clades evolving under non-extreme rates of change. However, simulation studies evaluating the accuracy of phylogenetic methods that explicitly treat error due to ILS do not always compare these methods to concatenation 30. Those simulation studies that do make comparisons to concatenation have not demonstrated uniformly superior accuracy of ILS-sensitive methods across all simulated conditions 31, and often use simple models of sequence evolution and/or few taxa 6, ^,^ 12, ^,^ 25, conditions that do not reflect typical empirical studies.

Furthermore, simulation studies concluding that concatenation is statistically inconsistent in the presence of anomaly zones and gene tree discordance often do not demonstrate superior accuracy by species tree methods under the same conditions 32, and are thus uninformative as to the practical merits of supermatrix and species-tree paradigms. Even when concatenation is compared to species-tree methods, it is not always specified whether apparent differences in performance are statistically significant 12; but see 33 and discussion below of other recent simulation studies that explicitly assess the accuracy of concatenation vis-à-vis species-tree methods.

Huang *et al.* 30 demonstrate how two sources of error - mutational variance and coalescent variance - impact the estimation of species trees using methods that account for ILS, but do not make direct comparisons to concatenation. Many empiricists still rely on concatenation for producing phylogenetic hypotheses for many taxa (e.g. ) and calibrating phylogenies with fossil information 34, ^,^ 35, ^,^ 36. Empiricists are likely to continue using concatenation for the foreseeable future, until methods that co-estimate genes trees and species trees *BEAST^6^ become more computationally feasible 37, and until truly synthetic and well-understood approaches to simultaneously account for the various sources of gene tree-species tree discord are developed. Thus, it is crucial that the relative accuracy of concatenation, vis-à-vis species-tree approaches, be explicitly delimited.

Herein we extend the experimental design of Huang *et al.* 30 to include traditional supermatrix methodologies, and explicitly assess the relative accuracies of species-tree methods and concatenation in estimating phylogeny. We find that the species-tree methods fail to outperform concatenation, and argue that, as a matter of best practices, studies involving methods sensitive to sources of gene tree-species tree discord should continue to compare results against those obtained by concatenation.

## Methods

Huang *et al.* 30 performed simulations to quantify the effects of mutational and coalescent variance in species-tree estimation. Specifically, they compared the performance of two methods – STEM 21 and MDC 16 – in resolving the true species tree under a number of conditions, but did not present comparisons to the supermatrix approach. Our goal was to explicitly make these comparisons. The relevant steps of the simulations performed by Huang *et al.* 30 involved 1) generating a species tree under a uniform speciation model, 2) simulating coalescent gene trees for each species tree, 3) simulating DNA sequences under a specified model of nucleotide evolution along the branches of each gene trees, 4) estimating gene trees from the simulated DNA matrix, 5) estimating species trees from the estimated gene trees using MDC and STEM, and 6) calculating the discord between the true species tree and the estimated species tree. Our simulations followed the same trajectory, except that we do not estimate gene trees separately and instead estimate species trees directly from concatenated matrices. Rather than rerun the simulations of Huang *et al.* 30 in full, we obtained from the authors their calculated measures of discord between results obtained for each species tree method and the true tree under a variety of conditions. We then compared these discord measurements to values that we produced by identically parameterized simulations analyzed under concatenation.

We obtained 50 species trees of eight taxa used by Huang *et al.* 30, which those authors generated in the Mesquite software package 38 under a uniform speciation model. We simulated 540 coalescent gene trees for each of the 50 species tree under a neutral coalescent model, constant population size, and no migration in the program *ms* 39 using the script provided by Huang *et al.* 30. For each of the 50 species trees, the simulations produced gene trees at total tree depths of 1N and 10N generations; these values reflect a range of total lineage duration. We used only the subset of all simulated trees with a single individual per species, in accordance with the common practice for supermatrix methods. For each gene tree, sequences of 1000 base pairs were simulated using Seq-Gen 40 under an HKY model of nucleotide substitution 41, a transition-transversion rate ratio of 3.0, gamma-distributed rate heterogeneity shape parameter of 0.8, and nucleotide frequencies of A=0.3, C=0.2, T=0.3, and G=0.2 for the ancestral sequence, in accordance with the model of evolution used by Huang *et al.* 30. These simulations assume a single clock-like rate of sequence evolution across the tree.

Huang *et al.* 30 evaluate the effects of increasing the number of loci in the data matrix on the accuracy of MDC and STEM in obtaining the true tree, measuring the accuracy of trees produced with matrices of 3, 9, and 27 genes. To make our supermatrix analyses comparable to those of Huang *et al.* 30, the 540 genes of each of the 50 species trees were concatenated into matrices of 3, 9, and 27 genes; for each species tree at each tree depth, this procedure produced 180 matrices with 3 loci (1N/3 loci and 10N/3 loci), 60 matrices with 9 loci (1N/9 loci and 10N/9 loci), and 20 matrices with 27 loci (1N/27 loci and 10N/27 loci). These concatenated data sets were analyzed in MrBayes 3.2.2 42 under the same model of nucleotide substitution used to produce the sequences (i.e. HKY), a uniform molecular clock model, and Dirichlet distributed nucleotide frequencies. MrBayes was run for each tree estimation until the standard deviation of split frequencies fell below 0.01. Estimating species trees using the substitution model under which they were simulated ensured that model misspecification was not an additional source of error in comparing method accuracy. After discarding the first 25% of the posterior as burn-in, we sampled every 100 trees from the posterior distribution to produce a majority rule consensus tree with all compatible clades added (using the “allcompat” command in MrBayes). The use of a consensus tree for subsequent analyses of discord between methods poses a potential problem, as calculations of topological discord (described below) may be biased in trees with polytomous clades. This potentiality is not addressed by Huang *et al.* 30, and their measure of discord was also based on the 50% majority rule consensus with all compatible clades. However, adding all compatible clades to the majority rule consensus ensures that most trees are fully resolved, and manual inspection of a representative subset of our estimated species trees found no polytomous clades. Encouragingly, recent research indicates that the majority-rule consensus is expected to minimize the discord between the estimated and the true tree 43.

Topological discord between estimated species trees and their true species tree was measured using the Robinson-Foulds (RF) distance 44 in the R package *phangorn* 45. The RF statistic measures the distance between two unrooted trees as the summation of the number of bipartitions unique to the first tree and the number of bipartitions unique to the second tree. At each tree depth, mean discord was calculated by averaging RF distances for each of the 180, 60, and 20 concatenated matrices associated with each of the 50 species trees. We then took the average of the means for all 50 species trees for each matrix size, such that the mean discord for matrices consisting of 3 loci, 9 loci, and 27 loci was estimated from a total of 9,000, 3,000, and 1,000 phylogenetic analyses, respectively. We note that Huang *et al.* 30 ran 20 independent replicates for each species tree at each permutation of tree depth and sampling design; we did not adopt this procedure here, as a single iteration of the simulations produced sufficiently large sample sizes. ANOVA statistical tests were used to evaluate differences in mean RF distances within and across methods using the values obtained by Huang *et al.* 30. We did not investigate whether clade support, rather than topological disparity, differed significantly between concatenation and species tree methods, but this topic merits further research. The scripts used to perform the simulations are deposited at http://dx.doi.org/10.6084/m9.figshare.1326720.

We acknowledge that eight-taxon matrices are significantly smaller than the matrices of most molecular phylogenetic analyses – though still larger than those used in many simulation analyses – and also recognize that STEM and MDC are not the most sophisticated ILS-sensitive species tree methods available. Our goal, however, was to perform a general test of the importance of considering concatenation when choosing among phylogenetic estimation methods. For the sake of comparability to Huang *et al.* 30, who seek explicitly to make recommendations regarding method choice, we did not expand their basic approach.

## Results

Comparisons among methods of a given matrix size demonstrate that mean discord values are statistically indistinguishable, with two exceptions: 1N/3 loci and 1N/9 loci (Table 2, Fig. 1). For 1N/3 loci, MDC performed significantly worse than the other methods under consideration, while at 1N/9 loci, STEM exhibited statistically superior performance. Within methods, increasing the number of loci results in a significant increase in accuracy for both 1N and 10N simulations (Table 2, Fig. 1). Across all simulations, concatenation is as or more accurate than either STEM or MDC, except at 1N/9 loci, where STEM appears, on average, to produce slightly less discordant trees than concatenation.

Under the simulated conditions, increasing the number of loci sampled improved accuracy across all inference methods, as is expected for methods that exhibit statistical consistency. Likewise, all methods showed increased accuracy for analyses of taxa related by longer effective internal branches, i.e. branches characterized by smaller ancestral populations, less ancestral polymorphism, and/or increased generational turnover (Fig. 1). Still, the RF values were surprisingly high at 1N, reflecting the inherent difficulty in confidently resolving recent divergences.

**Table 2. Results of ANOVA tests for differences in accuracy among methods (concatenation, STEM, MDC) with different numbers of loci and between methods with the same number of loci:** Accuracy measured by the topological distance (Robinson-Foulds distance) between the true and the obtained tree. Significant differences denoted with asterisks. See also Figure 1.

	3 loci	9 loci	27 loci	Concatenation	MDC	STEM
1N	<0.001*	0.0255*	0.6384	<0.0001*	<0.0001*	<0.0001*
10N	0.3601	0.8233	0.8505	<0.0002	<0.0001*	<0.0011*

**Mean and standard deviation estimated from a total of 9,000, 3,000, and 1,000 phylogenetic analyses for the 3 loci, 9 loci, and 27 loci analyses, respectively.**
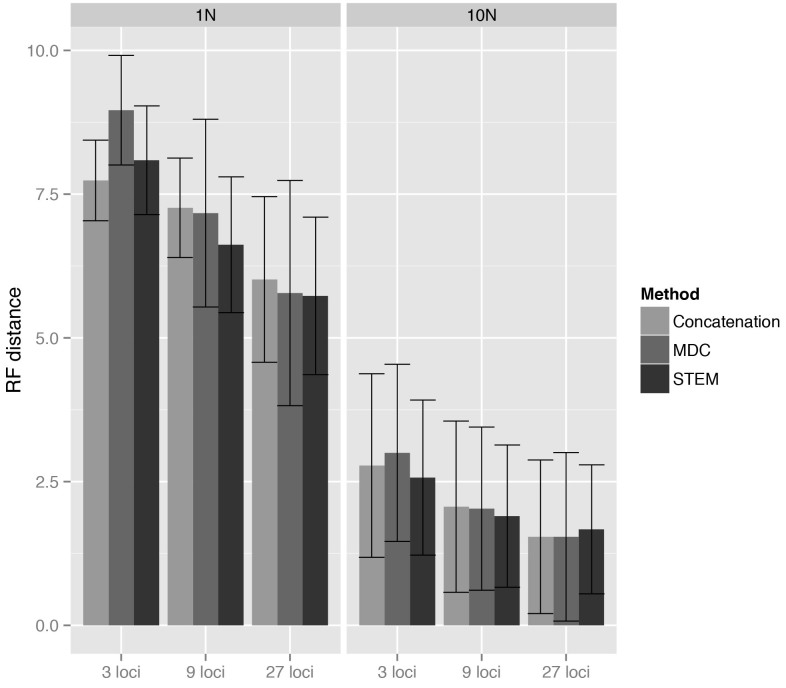

## The Phylogeneticist's Toolbox

Our simulations demonstrate that concatenation can perform as well or better than methods that attempt to account for sources of error introduced by ILS (Table 2, Fig. 1). This result corroborates some recent empirical findings that concatenation and species-tree methods can produce near-identical results 27, ^,^ 28, ^,^ 33, ^,^ 46, and is concordant with simulation studies indicating that ILS-sensitive methods do not consistently outperform concatenation under all conditions 31, ^,^ 33, ^,^ 46, ^,^ 47, ^,^ 48, ^,^ 49, ^,^ 50. Indeed, concatenation can outperform species-tree methods when levels of ILS are low, few loci are used, or gene trees have low phylogenetic signal 33, ^,^ 47, conditions that likely reflect the reality of many phylogeneticists working with mitochondrial and nuclear markers generated by traditional methods of sequencing to estimate large scale phylogenetic relationships 34, ^,^ 35, ^,^ 36, ^,^ 51, ^,^ 52, ^,^ 53. In addition, simulations by Patel *et al.* 49 and analysis of mammalian empirical data 50 indicate that concatenated analyses benefit from increased power vis-à-vis “shortcut” coalescence methods (i.e. those that estimate gene trees and species trees separately) when trying to resolve deeper divergences, especially when more loci are used. In these cases, high mutational error and gene tree heterogeneity are significantly more detrimental to shortcut coalescent methods than they are to concatenation, resulting in more accurate concatenation-based trees despite the incorrect assumption that all loci share a common topology. Binning techniques that use concatenation to produce “supergene trees” as input for coalescence-based analyses may improve the accuracy of species tree estimation by alleviating the bias introduced by using numerous small loci 54.

Gene tree-species tree discordance is a well-documented phenomenon, and we do not question that it poses a significant challenge to accurate inference of phylogeny; however, the growing body of research indicating that concatenation is not uniformly worse than discordance-sensitive methods should give phylogeneticists pause. That concatenation remains reasonably accurate under a range of conditions suggests that it retains practical utility. Where results from concatenation are concordant with those obtained from discordance-sensitive methods, the phylogeneticist can be reasonably confident that the resultant phylogenetic hypothesis is robust. Where concatenation and discordance-sensitive methods disagree significantly, the disparity may indicate regions of the tree in need of focused attention and might suggest possible biological factors confounding phylogeny estimation 55, or may indicate critically violated assumptions in one or both approaches 50. In these cases, morphological, physiological or ecological evidence may provide support for one hypothesis over the other.

Though many methods have been developed to account for error due to ILS (Table 1), other biological processes (hidden paralogy and horizontal gene transfer) can confound accurate phylogenetic inference; however, empirical researchers cannot know *a priori *the relative contributions of these processes to the genealogical history of a lineage, or to what extent discordance among gene trees may be due to methodological or sampling error (e.g. poor taxonomic sampling; model misspecification, species misidentification, long branch misplacement, arbitrary resolution of gene trees) 50, ^,^ 56. While concatenation has been rightly criticized for not accounting for known sources of phylogenetic error, shortcut coalescence methods assume that *all *error is due to incomplete lineage sorting – that is, that gene trees are known with certainty 50. To date, no species tree method simultaneously accounts for all sources of gene discordance 57; until well-understood, synthetic approaches to co-estimating gene trees and species tree and accounting for all sources of gene tree-species tree discord are developed, the continued use of concatenation has practical merit insofar as it avoids making *a priori *assumptions about the extent to which various sources of uncertainty have influenced the evolutionary history of the taxa under consideration. Thus, we argue that concatenation remains a useful component of the phylogeneticist’s toolbox as a “null” methodology against which results from alternative methods of inference can be compared and interpreted.

That concatenation remains an important part of the phylogeneticist’s toolbox is borne out by recent analyses indicating that the method exhibits greater power to overcome sampling error and discrepant patterns of homoplasy 50, and that “concatalescence” methods can make explicit use of concatenation in improving the quality of input trees prior to coalescence-based estimation of the species tree 54. Moreover, estimation of time-calibrated phylogenies combining molecular and morphological data (extant and fossil) is only possible in concatenation analyses, and concatenation has been shown to be more resilient to the effects of missing data than species tree methods 58, ^,^ 59.

We did not test improvement in accuracy of species tree estimation by using multiple individuals of the same species. New species-tree methods that have been developed to handle phylogenomic data of hundreds of loci have also used one individual per species when testing for accuracy and performance 46, a common practice in studies aiming to infer large-scale interspecific relationships 34, ^,^ 36, ^,^ 51, ^,^ 52, ^,^ 53. Although our results and the findings of phylogenomic studies indicate concatenation exhibits reasonable accuracy under certain conditions, we recognize that phylogenetic studies using either supermatrix or species-tree methods may benefit from including multiple individuals per species, especially for recent divergences 30, ^,^ 49, as the additional data help account for intraspecific allelic diversity when estimating interspecific relationships.

Research is only beginning to shed light on the relative accuracies of species-tree methods and concatenation for genomic-scale data matrices with thousands of taxa. Simulations indicate that coalescent-based methods improve accuracy for genome-scale matrices 33, though adding loci for which gene tree estimates are poor may be more detrimental for ILS-sensitive methods than for concatenation 49, ^,^ 50. Recent empirical research suggests that rapid radiations may make some internal nodes permanently intractable, regardless of methodology or sampling (e.g. few taxa and genes 60, many taxa and few genes 61, or few taxa and many genes 27).

The phylogeneticist’s toolbox continues to grow as our understanding of the dynamics of evolving populations improves, and the last year has witnessed the development of numerous methodological tools and considerations for practicing systematists. For example, recent research has produced posterior predictive model checking for coalescent models 62 and new methods of data exploration to reveal the causes and consequences of patterns of gene tree discordance 63, ^,^ 64. We do not dispute that the proliferation and refinement of new methodologies is crucial to the continued success of phylogenetic research. Our purpose here is to demonstrate the importance of explicit comparison of contemporaneous and classical methodologies, and to highlight that the addition of theoretically important parameters may not always produce practical gains. We conclude that concatenation exhibits statistically comparable accuracy under a range of sampling and tree depth conditions vis-à-vis some existing species tree methods, and urge molecular phylogeneticists to thoroughly evaluate the performance of methods that model gene tree-species tree discord against concatenation.

## Competing Interests

The authors have declared that no competing interests exist.
